# Audio Guide for Visually Impaired People Based on Combination of Stereo Vision and Musical Tones

**DOI:** 10.3390/s20010151

**Published:** 2019-12-25

**Authors:** Walter C. S. S. Simões, Yuri M. L. R. Silva, José Luiz de S. Pio, Nasser Jazdi, Vicente F. de Lucena

**Affiliations:** 1 ICOMP—Instituto de Computação, UFAM—Federal University of Amazonas, Manaus 69067-005, Brazil; zecapio@gmail.com; 2 UFAM-CETELI, PPGEE, and PPGI, Federal University of Amazonas, Manaus 69067-005, Brazil; 3 Institute of Industrial Automation and Software Systems, University of Stuttgart, 70550 Stuttgart, Germany; Nasser.Jazdi@ias.uni-stuttgart.de

**Keywords:** indoor positioning, data fusion, landmarks, Kalman filter, particle filter

## Abstract

Indoor navigation systems offer many application possibilities for people who need information about the scenery and the possible fixed and mobile obstacles placed along the paths. In these systems, the main factors considered for their construction and evaluation are the level of accuracy and the delivery time of the information. However, it is necessary to notice obstacles placed above the user’s waistline to avoid accidents and collisions. In this paper, different methodologies are associated to define a hybrid navigation model called iterative pedestrian dead reckoning (i-PDR). i-PDR combines the PDR algorithm with a Kalman linear filter to correct the location, reducing the system’s margin of error iteratively. Obstacle perception was addressed through the use of stereo vision combined with a musical sounding scheme and spoken instructions that covered an angle of 120 degrees in front of the user. The results obtained in the margin of error and the maximum processing time are 0.70 m and 0.09 s, respectively, with obstacles at ground level and suspended with an accuracy equivalent to 90%.

## 1. Introduction

Computational models of outdoor navigation have resulted in numerous applications [1]. However, the sensor adopted in the global positioning system (GPS) has substantial operating restrictions on indoor building structures owing to the presence of services that affect the satellite reception process [2].

Indoor positioning systems (IPSs) map a set of human activities indoors, such as locating, navigating, climbing and descending stairs, going through a door, turning left and right, and so on. Besides, the system must identify objects along a path, whether fixed or left at these locations [3]. Recognition of these human activities can provide a range of opportunities in location, navigation, and tracking services, as already available in outdoor environments [3].

Zengke and his co-authors developed an integration scheme of the global positioning system (GPS) with an inertial positioning system [2]. The authors’ observation was related to the range of GPS signals within building structures, making it challenging to locate and navigate a person safely. The authors used an integrated scheme for improving GPS signals to inertial sensor signals, with positioning error correction by the Kalman algorithm to improve the target position accuracy in the indoor scenario. In order to validate the method, an experiment was performed in a real environment, with the results compared with the separate approaches (GPS and inertial sensors). The average error was 1.70 m for the GPS, 0.89 m for the inertial system, and 0.42 m for the integrated model. The results indicate that the unified scheme corrected the positioning error and supplied the absence of GPS signals at some points of the building structure.

Several authors have begun to look for arrangements between different techniques and technologies to make IPS capable of providing accurate information in many types of scenarios [4,5]. The increasing complexity of indoor positioning models represents a new problem to be solved regarding accuracy and time, which must consider the limitations of the adopted device [6].

One way to define IPS is to consider the relationship between the level of accuracy and the acceptable time for information delivery. In simpler systems, linear mathematical formulations require less processing, but have error margins that need to be addressed by algorithms such as Kalman’s linear filter [7]. In nonlinear systems, the most complex calculations adopted have results very close to the reference values, but require more time and processing to reach these values, as seen in particle filter applications [8].

A common problem for navigation systems targeting the visually impaired is the identification of obstacles, either on the ground or vertically, which can cause accidents [4].

This paper proposes a solution capable of providing an audio guide on the location of an obstacle. The guide should inform the presence and position of obstacles regardless of the size and height of the perceived object. This proposal aims to overcome the limitations of traditional navigation models.

Finding a model that can perceive the objects, fixed or moving, arranged on the route is essential for delivering a reliable navigation guide. Therefore, the still open problem that needs to be addressed is as follows: how to provide more accurate and reliable information to visually impaired users about the horizontal and vertical position of an obstacle in indoor navigation systems?

Recent research indicates that hybrid indoor positioning systems, which combine data fusion techniques, allow their use in scenarios with different characteristics, but always facing, as one of the main problems, the search for the balance between performance and processing time [5]. Each algorithm and sensory approach has the advantages of its separate or combined application, requiring a thorough knowledge of its characteristics and the most appropriate context for its use [1]. Thus, the research hypothesis seeks to answer the research problem using hybrid navigation schemes.

Formally, this study presents an indoor positioning solution applied to the navigation context, which has different data and algorithms, giving the model a hybrid character, aiming to increase the final system accuracy, keeping the processing within a tolerable time for navigation systems.

Hybrid navigation is an arrangement that combines linear and linearized approaches to provide reliable and fast information. The route guidance technique implements greedy routing, which consists of choosing a geographical nearest neighbor with low processing consumption, representing the obstacle to be avoided [9].

From this hypothesis, iterative Pedestrian Dead Reckoning (i-PDR) algorithms and obstacle detection in 3D sound algorithms were developed. The i-PDR algorithm represents the association of the PDR algorithm with a Kalman linear filter and can consume a route received by the Dijkstra algorithm to decrease error margins iteratively as the system is used. Obstacle detection in 3D sound is an algorithm that combines stereo vision with a sound scheme in different musical tones to indicate the horizontal or vertical position of the obstacle.

Once defined and constructed, the i-PDR and obstacle detection in 3D sound algorithms by computational view were tested and evaluated. The i-PDR algorithm was compared to the pedestrian dead reckoning (PDR) algorithm, and the obstacle detection in the 3D sound algorithm was compared to approaches using ultrasonic sensors and computer vision. The functioning of the algorithms, as well as the analysis of similar processes, are presented in the next sections.

This paper presents the topic of safe browsing for visually impaired users for discussion, with the purpose of guiding users and generating audible alerts about obstacles. The approaches and results are formalized in this article in the following manner. Section 2 presents the strategies and outcomes of a set of related works to increase the understanding of the boundaries of the approaches chosen for this paper. Section 3 shows the proposed hybrid positioning system. Section 4 contains the protocol and setup of the experiment, as well as an evaluation of the experimental results. Section 5 presents the final arguments and conclusion of this paper.

## 2. State of the Art

Visual positioning systems use images captured by one camera (monocular model) or multiple cameras (stereo image) [10]. Monocular models are faster, but do not provide distance and depth information. Stereo imaging systems provide information about the distance between cameras and objects, but generate a large amount of data [11]. To use stereo vision in navigation applications, strategies that remove spurious data must be applied to strike a balance between level accuracy and processing time.

Xue presents a study on the reduction of indoor mapping time so that this operation does not impede the use of indoor positioning systems [12]. In this study, maps use information extracted directly from video streaming, reducing samples to a set of 100 images per location by the random sample consensus (RANSAC) algorithm. The speeded-up robust features (SURF) algorithm was used to build markers similar to Haar’s model quickly, disregarding angle and lighting variations. The accuracy level obtained in the tests was 70%, resulting in a maximum error distance of 2.00 m for the location informed by the system.

Systems based on visual recognition also have limitations, such as lighting, angulation, and having to deal with a large amount of data and have a high computational complexity [11]. Many authors have chosen to increase the number of sensors and associate different perspectives on data to make the system more robust, making positioning information reliable for the user [13]. Approaches that relate the use of two cameras bring a 3D perspective of the scenario and improve the perception of elements, including distance information through depth maps [14].

Chen associated visual perceptions with inertial information in his hybrid navigation system through a Kalman filter (KF) to obtain a 3D perspective of the scenario [6]. The KF algorithm also reduced the accumulated errors presented by the PDR algorithm during navigation. A total of 937 landmarks were set at 61.00 m, and a small robot built for testing traveled the designated path, observing the coordinates obtained along the course. Error margins averaged 0.40 m compared with the default values recorded for each location.

Presti and his co-authors dealt with the visual location of obstacles close to the user (usually about 1.00 m away), which are usually perceived by the use of walking sticks [15]. The system called Watch Out provides information about the presence and position of obstacles to the user through polytonic sound messages, emitted in three volume ranges (low, medium, high). The low tone indicated distant obstacles (above 2.5 m), the medium tone indicated obstacles at a medium distance (between 1.5 m and 2.5 m), and the high tone indicated obstacles at close range (below 1.5 m). All tests were user-centered to improve and evaluate the sonification technique. Thirteen users with low vision were invited to participate in the testing process and questionnaires. The results show that the system is considered usable and can guide users to avoid more than 85% of obstacles.

Massiceti and her coauthors developed an inland navigation model based on the relationship between visual perceptions and a sound relative to the position indicating obstacles or a target [16]. The authors extracted 3D visual spatial information to enable the mobility of blind and visually impaired people. The study followed a model similar to echolocation, modulating the volume according to the distance between the user and the obstacle. The authors built a virtual maze so that user decisions generated data to predict behaviors in a real scenario. Test participants had to complete two tasks: navigate the maze and dodge obstacles, with the task completion time and the number of collisions used as indicators of successful navigation. Users using only sound information were 65% slower than users using sound information associated with the central guide generated by computer vision. Modulating tinnitus volume proved to be 20% faster than spoken information.

Bujacz also used the relationship between stereo vision, ultrasonic sensing, and the activation of modulated sounds at different frequencies to indicate the position of obstacles placed along the way [17]. The authors were inspired by sonar-based models to provide feedback to the user, using a five-tone full-scale sound bank and two octaves of these tones played by a piano to represent certain positions of the test scenario. Each sound was played for 0.2 s and was triggered about 1.5 s before the user collided with the obstacle.

The solutions discussed in this article were organized to highlight their characteristics, such as proximity methods, PDR, and Simultaneous Location and Mapping (SLAM) (Table 1).

Contrary to the works discussed, this paper presents a solution that uses only cameras as a physical resource and a sample reduction and data fusion algorithms. The sample reduction algorithm selects the most representative data captured by the stereo vision system and cast into a depth map. The fusion algorithm performs data prediction and correction operations without camera triggering to reduce processor and runtime consumption, while keeping the system reliable.

The proposed system combines visual data through autonomous mathematical approaches to deliver the results to a combination of pedestrian dead reckoning (PDR) and Kalman algorithms.

The sensors used to obtain environmental information are of the relative type, and the consumed data do not require the use of external sensors, such as systems that use mathematical approaches to estimate value [10,11]. The following sections detail each step.

## 3. Proposed Indoor Positioning System

This paper presents the development of a navigation architecture that provides information about the user’s location while navigating in an indoor environment. For the visually impaired audience to be served, the instructions provided by the system during navigation are natural, following the model similar to human cognitive navigation.

In addition to the obstacle identification process and the virtual markers defined between obstacles, some aspects were taken into consideration for decision making in the construction of indoor navigation architecture, such as the following:

1. Security: The application is local and embedded. Only the device calculates and processes location information, ensuring data access privacy;
2. Delay or Response Time: Choice of techniques that provide acceptable time for visually impaired navigation;
3. Robustness: The choice of prediction and correction techniques makes the system less fault-prone than using readings directly, and reduces the need for processor utilization, reducing response time to a tolerable limit;
4. Complexity: as a criterion, we chose the sample reduction of data accompanied by data fusion and the emission of sound messages as output from the system;
5. Limitations: Navigation algorithms use probabilistic methods and require constant updating of positioning data to inform the user.

**Figure 1:**
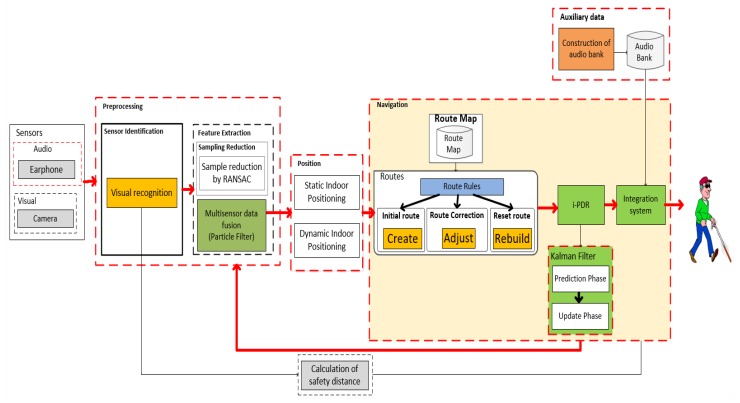
Indoor navigation system architecture. RANSAC, random sample consensus; i-PDR, iterative pedestrian dead reckoning.

The proposed architecture assumes that the system must be fast enough to provide reliable information for visually impaired users. The strategy used to reduce processing time was sample reduction and merging of the resulting data to indicate viable paths and obstacle positions, as illustrated in Figure 1.

The proposed system architecture shows the application of sampling algorithms before the merge process to reduce the time spent in data processing, in addition to maintaining the correct location level. Each subsystem adopts its method of data manipulation to construct a final representation according to the indication of gain of its use in a decision-making process. Each step is detailed below, indicating its methods and algorithms.

### 3.1. Preprocessing

Data preprocessing is the step necessary to perform the interventions on the raw data and achieve the expected standards for the definition of the markers. The purpose of manipulating data before sending it to redundant and complementary fusion algorithms is to maintain only data classified as strong (quality) and to reduce the time required to recognize the pattern used in the received data.

In general, the flow of this step indicates two main tasks: the identification of the sensor type, to direct the data to the appropriate algorithms, and the characteristic extraction algorithms, which consume the data and trigger the fusion algorithms, as detailed below.

#### 3.1.1. Feature Extraction

The extraction of characteristics has two functions: sample reduction used in the preparation of the mapping, and multisensory data fusion, which applies linear processes to deliver information in the navigation process.

Resource extraction uses a data set obtained from sensor type identification and applies a weight-based sort order to define a sample subset of relevant resources.

#### 3.1.2. Sample Reduction

The strategy used to reduce processing time while maintaining the robustness level of the marker was to select representative samples of raw data. The algorithm for this data decrease for a robust sample was the probabilistic model called random sample consensus (RANSAC).

RANSAC is an iterative process algorithm that allows robust adaptation of a model *y* =f(x;α) to an S data set, containing inliers and outliers [11]. The least squares (LS) method estimates a reduced model, observing data classified as inliers, solving up to 50% randomly chosen outliers [11]. The RANSAC algorithm has its operation defined in five steps:

Estimate the parameters $\alpha_{tst}$ of a randomly sampled subset of n data points of S;

Determine the subset of inliers $S_{tst}\subseteq S$ to be the set of data with distance t for the model;

If this set of inliers is the largest at this point, make $S_{IN}=S_{tst}$ and e $\alpha =\alpha_{tst}$;

If $\left|S_{IN}\right|<T$, where *T* is some threshold value, repeat steps 1–3, otherwise stop the algorithm. The stopping criterion is given by a maximum number of iterations $T_{max}=40$;

After *N* trials, finalize the process.

A threshold value, established at random, restricts and adds the amount of data held to the inliers collection. This process is iteratively triggered until it reaches the highest value, considered here as the best sample set from the original data set [12].

#### 3.1.3. Multisensor Data Fusion

Data fusion by the particle filter (PF) represents the application of a particle filter to the data and the fast delivery of the processed values to the i-PDR navigation algorithm.

The particle filter has recursive functions so that variables of interest representing the most robust data are used during navigation. In each iteration, all particles change according to the existing model (prediction stage) and last reading data (update stage) [8]. This process reduces the low representativeness of the data. The four steps of applying the particle filter are as follows: propagate, update, estimate position and orientation, and resample the data.

The propagation step corresponds to the position update phase for each particle. Both step length $l_{t}$ and direction orientation $\theta_{t}$ are passed through the PF component. Once received, the step length and direction orientation are treated as data modified by Gaussian random noise. Hence, the new location and orientation direction of the particle *i*th at time t are as follows:

(1) $$\theta_{t}=\varphi_{t}+\varepsilon ,$$

(2) $$x_{t}^{i}=x_{t-1}^{i}+\left(l_{t}+\delta \right)\cos\left(\theta_{t}\right),$$

(3) $$y_{t}^{i}=y_{t-1}^{i}+\left(l_{t}+\delta \right)\sin\left(\theta_{t}\right),$$

where δ is the Gaussian noise for the length of the pass, and ε is the direction.

Measurement updating is the phase responsible for correcting the weights that each propagated particle presents. First, the particles are collected during time *t*. Particles that deviate from the most representative group are neglected. The other particles indicate the most likely location. Particle weights are updated by the following equation:

(4) $$w_{t}^{i}=\frac{w_{t-1}^{i}}{\sum_{i\in P_{t}}w_{t-1}^{i}}.$$

The center of all particles is calculated and compared to the previously estimated positions to indicate the position and orientation estimate.

A simple decision-making process verifies whether the number of particles has exceeded the maximum amount allowed to direct to the resampling operation or if it is still below the limit and can thus perform another verification of the data sampling.

The resampling step first eliminates particles with weight equal to zero. All the surviving particles generate a new filtering process under Gaussian distributions with their weights. The weighted center of all particles provides the estimate of the user’s current position.

### 3.2. Position

In this work, the depth map generated by stereo vision was defined as the object’s location strategy, without the need to build classifiers, which use a lot of processor and memory.

Unlike strategies such as that of Skulimowski and his coauthors, who treated spatial information captured by stereo vision as a 3D matrix, this paper presents an obstacle perception algorithm that decomposes the 3D matrix into two 2D matrices that represent the ground and the information positioned vertically in front of the user [18].

#### 3.2.1. Static Indoor Positioning

**Figure 2:**
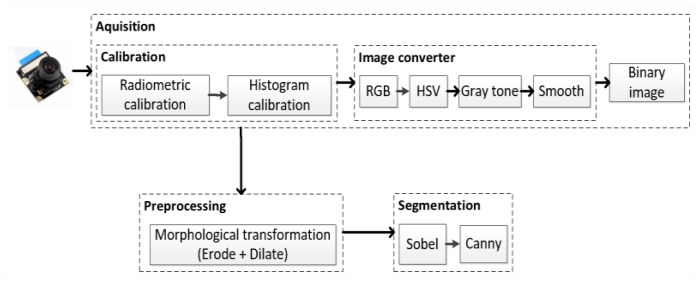
Visual marker recognition scheme.

The visual localization system requires that some steps be performed, such as acquisition, preprocessing, segmentation, and resource extraction [10]. Then, the interpretation of the information generated by the computer vision algorithms inserts a coordinate code to be used during navigation. The visual recognition scheme is given in Figure 2.

The acquisition stage represents the reception of images that undergo radiometric and histogram calibration treatments for normalization of illumination and brightness contrasts, and to automatically adapt the system to new angle conditions [6].

In the image conversion phase, Red, Green, Blue (RGB) images are converted to HSV format. HSV separates information into its hue (H), saturation (S), and value (V) components, which make it easier to identify objects in images [10]. HSV images are then transformed into grayscale to reduce the system processing cost. Finally, grayscale images are transformed into an integral image, which is the least complex binary representation possible [12,19].

Preprocessing represents the application of morphological transformation and smoothing algorithms to reduce the amount of noise [10]. Morphological transformation combines erosion and expansion algorithms that follow a minimum neighborhood requirement [4].

In the segmentation stage, the combination of Canny and Sobel filters is applied, reinforcing the characteristics of visual objects over the other elements of the images. The image resulting from the segmentation phase is delivered for extraction and recognition [10].

The Sobel filter checks the internal continuities of objects by the gradient intensity at all points in the image [20]. Mathematically, the Sobel operator calculates the approximation of the horizontal and vertical derivatives of the original image for two reference matrices with a 3 × 3 dimension [21]. The Canny filter checks the continuity of the outer edges of objects [20]. The Canny filter looks for the minimum and maximum value within the sample space to find the shortest distance that connects two points by approximating from the first Gaussian derivative.

**Figure 3:**
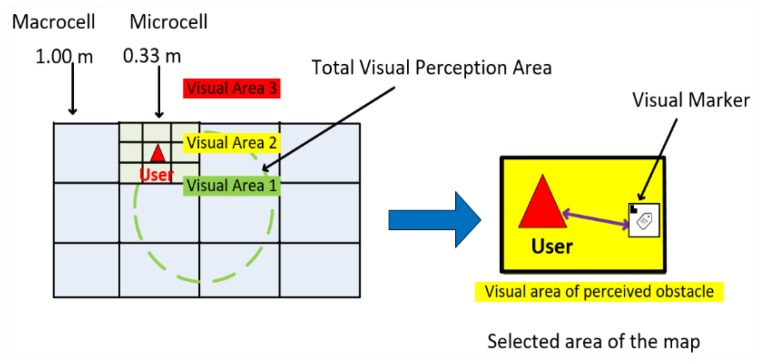
Target tracking scheme.

Hybrid localization combines visual information into a complementary fusion by Kalman’s linear filter algorithm, correcting any localization errors indicated by applying a single camera reading. It is still possible to combine map matrix cells to enlarge the macro-location area or subdivide a micro-region to increase the perception of each location. Figure 3 shows the target location scheme.

**Figure 4:**
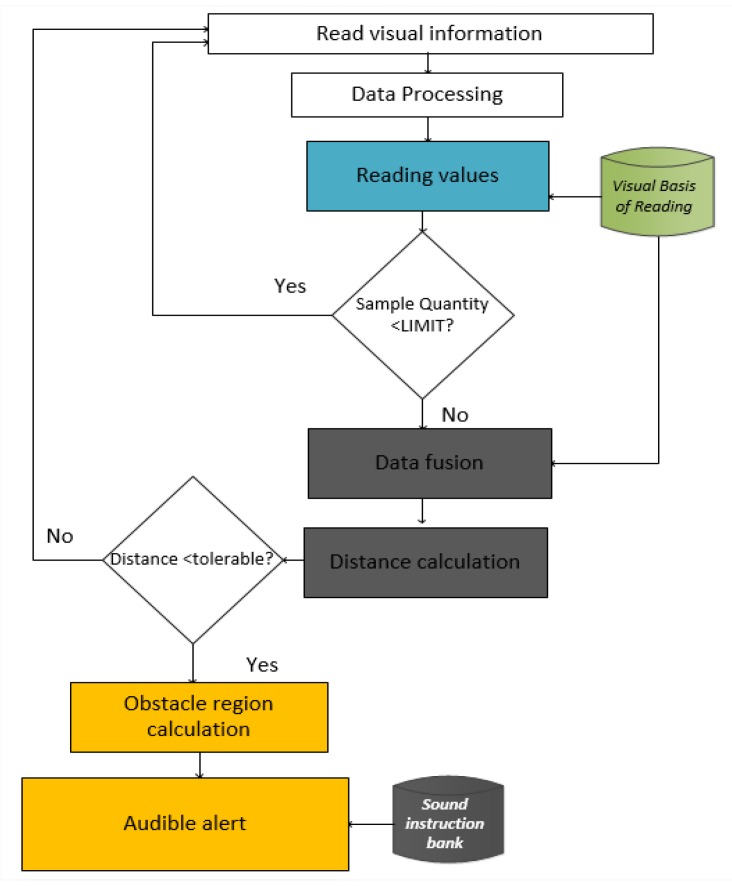
Construction of visual and hybrid information.

The behavior of the algorithms, involving their data selection, sample reduction, and location representative construction processes, is shown in Figure 4.

For an acceptable relationship between accuracy level and processing time, a set of linear algorithms has been built to use less processor and memory and generate the least possible impact [11]. These algorithms are triggered from the receipt of data until the delivery of the results and execute the decision making, which maintains or discards data according to the error control indicators [22].

#### 3.2.2. Dynamic Indoor Positioning

Linear and linearized algorithms were constructed to follow the target during its displacement (guided navigation), correcting eventual errors by the continuous updating of its position by comparison with the reference registers saved on the map.

Computer vision-based approaches provide information about the distance from the start to the end of a route more broadly and are also able to provide more subtle information needed in situations such as turns and orientation changes while navigating the route.

**Figure 5:**
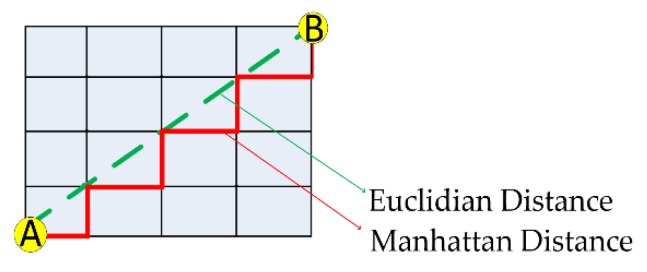
Components of target-tracking algorithms.

The straight distance is obtained by applying the Euclidean distance (Equation (5)), and the distances containing curves are obtained by using Manhattan distance (Equation (6)), as shown in Figure 5.

The hybrid approach needs to be robust and fast, so it uses combinations of data and algorithms so that user and obstacle tracking is continuous and close to the real value of the user’s location. Hybrid localization algorithms make a simple decision about the data received from the sampling step, discarding most outliers that may contain noise and contaminate the result, as described by Alatise [5].

(5) $$M_{p}=\frac{p_{1}.x_{1}+p_{2}.x_{2}+\cdots +p_{n}.x_{n}}{p_{1}+p_{2}+\cdots +p_{n}},$$

where $M_{p}$ is the weighted arithmetic mean; $p_{1},\;p_{2},\;\ldots ,\;p_{n}$ are the weights of the computational vision markers; and $x_{1},\;x_{2},\;\ldots ,\;x_{n}$ are the data values obtained by the locations of the positioning systems.

### 3.3. Navigation

The indoor positioning system (IPS) runs directly on the wearable device, to be always available and to provide the information within a tolerable time.

A rule represents, in a macro way, what should be done when certain situations are perceived. A procedure represents the following set of steps to achieve the result.

The processing of the subsystems based on computational vision sensors occurs in parallel, and their results provide the basis for the hybrid location formation.

#### 3.3.1. Route Rules

The heuristic used to define the route between reference points was to use an absolute and interval location model, adding the costs associated with the arcs in a polynomial problem. Dijkstra’s algorithm makes it possible to apply heuristics by linking the location records in an adjacency matrix [9].

In traditional route definition models, it is common to use markers to establish neighborhood relationships and allow the user to provide guidance [9]. In this work, the markers represented the positions of the obstacles, to be used as a reference in identifying the viable path, thus allowing safe navigation and low risk of accidents (collisions). Therefore, the adjacency matrix uses the values of 1 and 0 to indicate whether the markers have a neighborhood relationship or not.

As with every route process, two positions are essential: the beginning and the end. All other intermediate localizations are calculated based on the neighborhood scheme and the processing cost criterion [23].

A reference route was built to record the positions available between obstacles and to be used to calculate error margins obtained by users. The guarantee that the start and end positions were the same value was to position the users over the start marker and for a support team member to position themselves over the final test marker. During guided navigation, the algorithm adjusts its position when the margin of error is within the 1.5 m limit. Above this value, navigation is interrupted, and the route is rebuilt.

#### 3.3.2. Guide

The navigation guide has the function of relating the user’s movement perceptions, indicating the route and the presence of obstacles and the delivery of the sound instructions to the user.

The guide module consists of the iterative PDR (i-PDR) navigation algorithm and the integration system, which calculates the position to be reached by the user in his displacement and attenuates the effects of the route deviations.

The i-PDR modifies the PDR by inserting an iterative update function. This intervention in the PDR algorithm was necessary to treat two main factors: velocity oscillations and location identification variations, which generate errors that accumulate along a trajectory and create position divergences when compared with the referential map register [22]. The i-PDR algorithm is iterative because it resets the PDR on each marker found, reducing the impact of accumulated errors present in the traditional PDR, as described by Koo and his co-authors [22]. The i-PDR represents the union of the PDR to the Kalman filter, which gives iterative and adjustment characteristics to the guiding algorithm.

The i-PDR begins its user position adjustment after the initial location, where the obstacles placed in front of the user are perceived as well as the viable path between these obstacles. The i-PDR utilizes preprocessing step data fusion and dynamic positioning, which considers angles and travel speed, to identify the user’s current position.

Candidate locations are indicated by the application of the K-nearest neighbors algorithm (KNN), which calculates the approximation of the reported navigation value to one of the values recorded in the adjacency matrix previously captured during navigation.

Owing to a large amount of data presented as candidates for location indication, only *k* reference points (RPs) are chosen according to the first *k* minimum distances identified by Equation (6).

(6) $$\left(\hat{x},\;\hat{y}\right)=\frac{1}{k}\overset{k}{\underset{t=1}{\sum }}\left(x_{t},\;y_{t}\right),$$

where $\left(x_{t},\;y_{t}\right)$ is the RP coordinate at time t, and $\left(\hat{x},\;\hat{y}\right)$ is the estimated coordinate of the observed point. The parameter q has two possible values, which are 1 to use the Euclidean Distance equation and 2 for the Manhattan distance equation. The construction of the hybrid marker takes the parameter *q* with a value of 1.

Assuming that the number of reference points (RPs) and the number of location markers (LMs) are *m* and *n*, respectively, the distances are defined as follows

(7) {Lqi=(∑t=1n|st−sit|q)1q,i=1,2, …, m,

where $s_{t}$ is the sample collected to identify the location at time *t* where the data are compared, and $s_{it}$ is the sample collected from the data received from the visual models.

All information regarding user displacement is recorded and used in the integration algorithm, which feeds the i-PDR algorithm and the Kalman linear filter. These algorithms provide the next position to be reached in navigation, applying adjustments necessary to decrease the error margins. Errors treated by the Kalman filter are systematic types, which occur within a pattern, and not systematic when they are without any established pattern [11,24].

**Figure 6:**
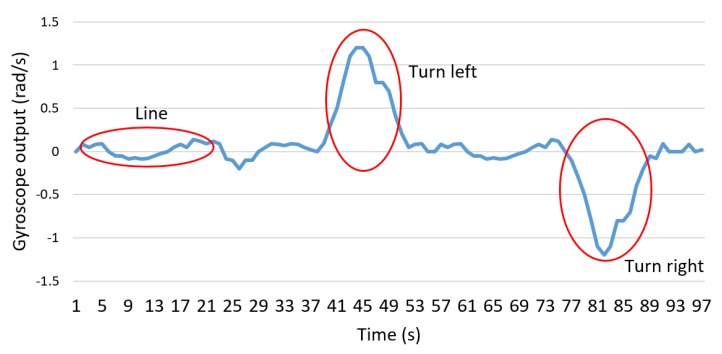
Identification of curves and lines.

Recognition of curves and straight lines results from the fusion of visual data using edge filters and a Gaussian filter [3,10]. This visual information relationship scheme identifies the direction of the curves (left and right curves), as shown in Figure 6.

### 3.4. Obstacle Identification

The integration module combines the processed guide data with the received obstacle detection data, indicating to the user the perception of obstacles through a sound scheme. The algorithms responsible for identifying the horizontal and vertical position of obstacles trigger sounds in the right or left ear, or both. The sound emitted is delivered to the user with higher or lower frequencies to indicate the presence and proximity of the obstacle, providing spatial reasoning.

The position of an obstacle can be calculated by identifying the object or by indicating the distance. In systems that use object identification, it is necessary to develop a classifier construction step and their mapping in the scenario [10]. On systems using distance indication, algorithms calculate the area where it has the most significant volume of data, indicating the presence of an object, whether this processes 2D (only perception of the ground level) or 3D images (ground level and vertical) [25]. The model adopted in this paper identifies the presence of obstacles by applying image processing without the use of classifiers, in two 2D matrices representing the horizontal and vertical planes.

Cameras are affixed at a similar distance from human eyes to apply a disparity calculation, which allows the difference between the two blocks of visual information to be identified by a matching function. The corresponding function arranges the right image into sample blocks to search the left image. The search process begins with minDisparity and moves left to the numDisparity value or finding the corresponding block [25].

The distance obtained between common reference points in two images is called disparity and gives you more detailed information about objects [11]. From the triangular relationship, it is possible to calculate *Z* as follows:

(8) $$Z=f\frac{b}{d},$$

where $d=x_{1}-x_{2}$ indicates the distance between the right image and the left image, and *d* is the pixel size. Equation (8) also allows calculating the distance between the user and the obstacle in front of him.

**Figure 7:**
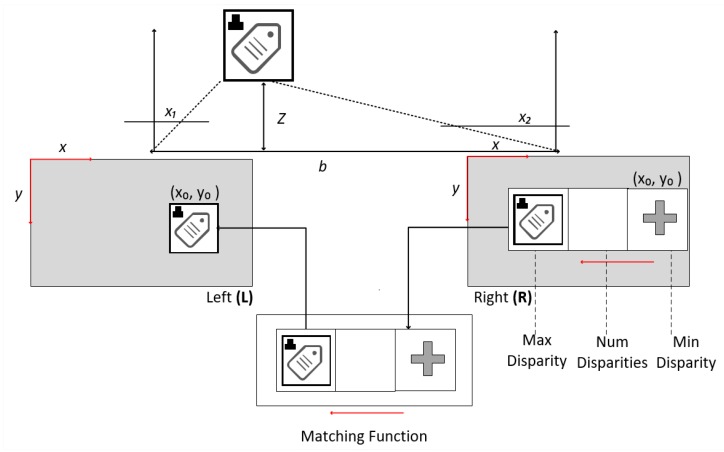
Systematics of the disparity map operation.

Objects close to the observer have a more significant disparity, whereas distant objects show less difference, as shown in Figure 7.

The function used in the project to calculate the disparity map is the FindStereoCorrespondenceBM of the OpenCV library, which uses block-matching methods and absolute difference sum (SAD) to find matching points between two images. The algorithm uses the preFilterSize function to normalize the brightness and texture of the images before the matching process in the two images.

The SAD algorithm applies the SADWindowSize function to match horizontal lines, eliminating mismatches by using a predefined number of pixels in the numberOfDisparities parameter. The preFilterCap function determines the center pixel of the images and serves as a guide for combining the two images [6].

The FindStereoCorrespondenceBM algorithm is based on block matching, which associates blocks and edges with information that generates high disparity [24].

The speckeWindowSize and speckleRange functions parameterize the size of the search window and the maximum acceptable variation to decrease the disparity [24].

### 3.5. Auxiliary Data

Distances perceived by the stereo vision system were associated with a sound scheme, where obstacles seen at ground level or suspended receive a distinct sound and frequency. This arrangement allowed the 3D perception of the environment, as indicated by Song in his acoustic localization system [26]. The activation of alerts for obstacles placed on the ground occurs when the distance is between 0.0 m and 4.00 m. For vertical obstacle identification, the maximum range is 2.50 m. The activation of the vertically arranged obstacle warnings occurs by evaluating the result of the distance between the user and the obstacle, up to the limit of 2.50 m.

**Figure 8:**
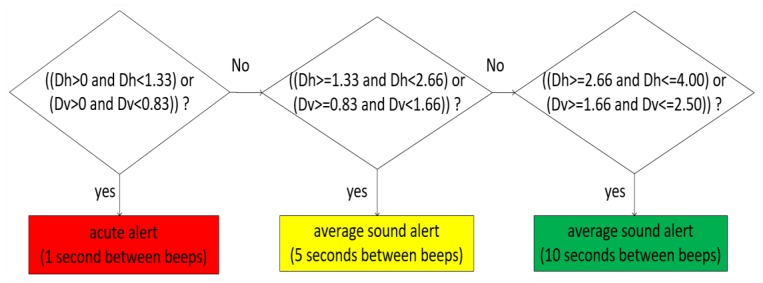
Sound alert scheme based on obstacle distance.

Music notes A + and C + alert the user to the presence of the obstacles using the following scheme:

- Note A +, which has a frequency of 440 Hz, shows horizontal obstacles (ground);
- Note C +, with a frequency of 264 Hz, shows vertical obstacles (floor to ceiling);
- Each note is played in three octaves (bass, medium, treble) to express near, mid-distance, and distant elements;

This model has received an audible alert frequency reinforcement using a universal obstacle perception language, where the speed of the audible alert emission is related to the user’s proximity to the obstacle. In the range where the sound is low, the interval between alerts is shorter and the time increases according to the music track. This warning sound language is similar to that used in the vehicle’s rear warning system, but is enriched with information about the obstacle height (vertical position), as indicated in the flowchart in Figure 8. The distance between the user and the obstacle is perceived from the horizontal (Dh) and vertical (Dv) perspective.

**Figure 9:**
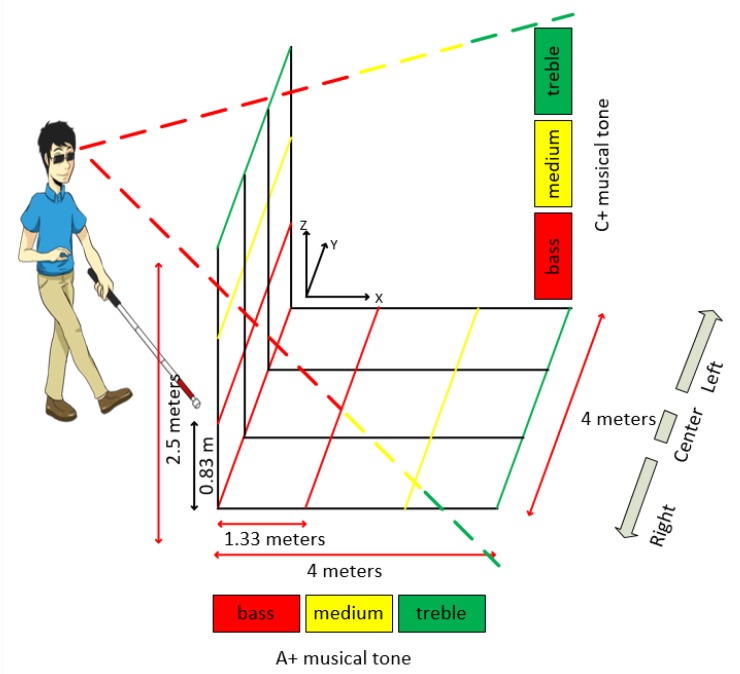
Obstacle detection scheme using stereo vision and audible alerts.

The audible alert system also indicates obstacles that interrupt the passage or obstacles that allow the user to circumvent the object so that the walking is not interrupted. The 3D sound location of an obstacle is also coded in relation to the user’s head position. Obstacles located on the right will be heard in the right ear, and vice versa. For example, if a small obstacle is perceived by the visual system, located in front of the user, close to their feet, a bass A + and C + sound will be played in both ears quickly. Figure 9 shows this relationship between the positions of the obstacles (horizontal and vertical), their distances to the user, and the sound alerts issued.

The emitted sound is digitally produced to standardize the sound frequency and to prevent the execution of an external media, which requires IO (input/output) operations and consuming processing, memory, energy, and especially time.

The audible alert system plays a key role in ensuring user safety in the perception of obstacles, but does not provide information on how the user should move in the environment. This way, the sound system receives a set of six short sentences of spoken sound instructions. This reduced amount of instruction was intended to reduce the learning time of using the system. The use of spoken instructions is intended to alleviate the user’s doubts about the action to be taken, as described in Zhangaskanov’s approach, through two short phrases indicating “high ground obstacle” and “low ground obstacle”, and the approach described by Spagnol, who used a bubble triggering scheme in a container containing liquid [27,28]. The six spoken instructions and actions to be taken by users are shown in Table 2.

## 4. Evaluation

A protocol that standardizes a set of procedures is designed to standardize testing and reduce the number of interference with results. The protocol describes the following steps:

- The user must perform a complete turn in the scenario without using the device to evaluate the impact of the audio guide on the perception of obstacles;
- The user must perform a complete turn on the scenario with the device;
- At the end of each test run, the user should be interviewed to record their usage perceptions.

**Figure 10:**
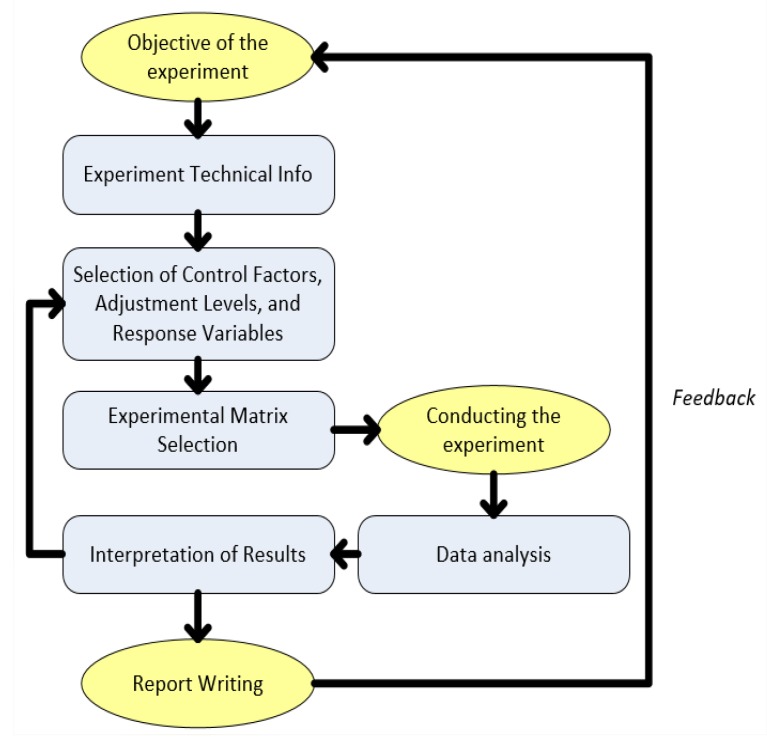
Protocol flowchart adopted for testing.

The protocol also describes how data, algorithms, and evaluations of physical devices should be performed. This concern about recording the technical part of the process allows us to observe the factors (variables that can be controlled) and the responses (process output variables), so that the experiment can be replicated. The block diagram of Figure 10 represents the scheme adopted in the investigation.

The factors observed in this experiment are the edge and mass information of the obstacles, as well as the position correction processes provided by the learning algorithms.

A scenario was used for tests containing straight corridors, turns, and the presence of obstacles. A set of objects along the way was an obstacle to evaluating the information provided by the navigation algorithm. The algorithm should calculate the distance and position of obstacles for the user and provide a viable path for use. The distance calculation results indicated by the mono and stereo vision algorithms were recorded for further evaluation.

The user test ends with an interview to record their perceptions of the system as a whole (physical and logical). The answers complement the thesis by inserting information about usability aspects to understand the target audience better and have indications of where to act to make the system more compatible with the expected demands.

The environment chosen to evaluate the prototype and algorithms was the Intelligent Environmental Laboratory (Amilab), belonging to the Center for Electronic Research and Development of Technology and Information (CETELI), of the Federal University of Amazonas (UFAM). The AmILab has dimensions of 10.83 m × 13.80 m, containing straight lines, curves, and obstacles.

### 4.1. Device Setup for the Experiment

A wearable mobile device was built to perform the tests. This device includes the pair of cameras and the central unit capable of processing the data and providing the information to the user. The mobile device considers the hands-free condition, which is an essential requirement in experiments with visually impaired people [4].

The prototype is constructed in white Acrylic-Styrene-Acrylonitriles (ASA) resin eyewear format, weighs 220 g, and has a size of 30 cm. The glasses have a support base over the nose and ear rods. The glasses are equipped with two RGB cameras with five-megapixel resolution and a 60 degree field of view, a 128 × 64 pixel transparent mini display, and headset. A Raspberry Pi 3 mini PC locally processes the data and sends the results from an external drive to draw the user’s offset in a graphical representation. An ASA resin printed box holds the processing unit and battery of 10,000 mAh (milliampere/hour) and 37 Wh (Watt/hour).

### 4.2. Experiment Setup

The experiments followed the predefined protocol and, in each test, the algorithms were evaluated alone and combined (hybrids) to record performance evolutions (precision versus time) and meet tuning needs.

**Figure 11:**
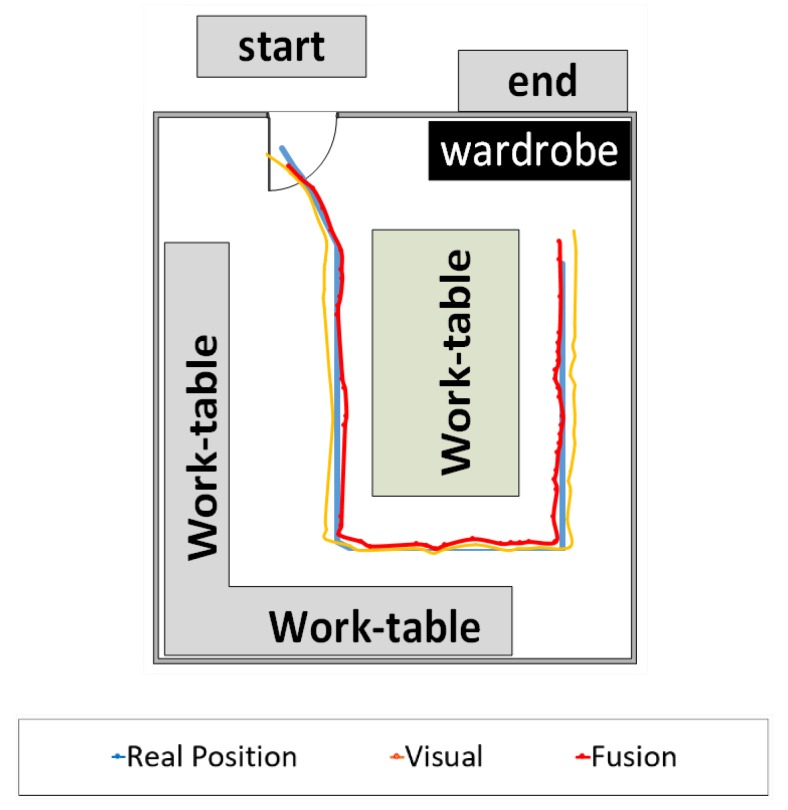
The result of target navigation in the lab.

The location test used an array of 462 cells, 0.33 m^2^ in size, to record the values of the navigation system locations, inserted into a 2D scenario schema (Figure 11). In this representation, the blue line indicates the reference points, an orange dashed line shows the positions of the visual location, and a solid red line shows the hybrid location.

The positions shown by the visual system differed from the expected values, mainly caused by natural light and the numerous intensities of artificial lights. These variations confused the interpretation, sometimes indicating the walls as part of the floor. The system presented an error rate of 11.3% when only one of the cameras (monovision) was triggered for ground-level perception, where interference caused by lighting variations was perceived. The data combination of the two cameras in the stereo model allowed a 3.60% error reduction.

To increase the level of accuracy, the hybrid model has a decision layer that holds or removes data within predetermined error limits. The fusion algorithms provided a more robust horizontal and vertical orientation perception, with an average error of approximately 0.186 rad every 10.00 m. Table 3 shows the average distance from the perceived margin of error during testing.

One criterion adopted in systems evaluation is the time factor. This criterion was chosen in each subsystem and in the final process to indicate the evolution of time for each physical and logical intervention, as shown in Table 4.

The time gain in the hybrid localization process was obtained by the perception of the standard deviation, which combined the data collected by the two cameras after the sample selection and calculated the position of obstacles.

Another criterion used to test and analyze the performance of the visual positioning system was to identify the number of frames processed every second (FPS) for each technique added to the process. The efficiency ratio is directly related to the number of frames delivered, where the higher the value, the more natural the passage of location information to the user during the navigation process. Table 5 shows the values obtained throughout the tests and the inclusions of the image processing techniques.

The i-PDR algorithm presented a mean localization error of 0.11 m and a direction error of 1.8 degrees, keeping the system stable in both positioning modes (static and dynamic). The hybrid model still had a 93.23% lower margin of error than the Wi-Fi-based system, which had the worst result among subsystems.

**Figure 12:**
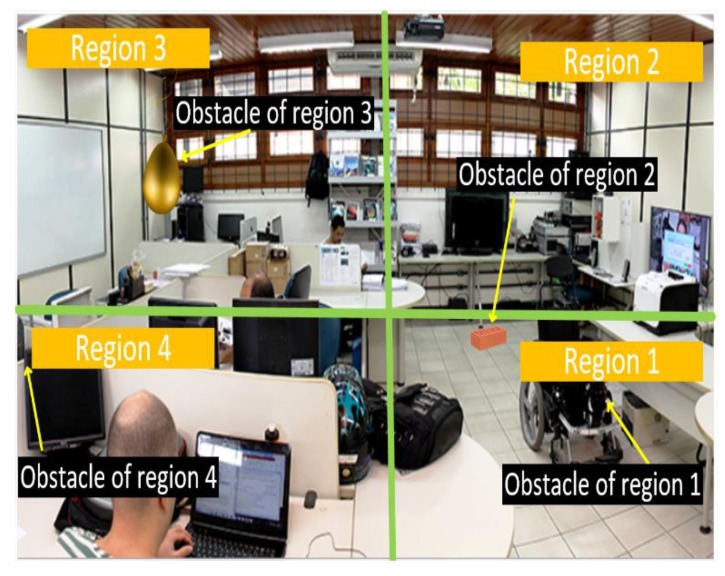
Visual markers under different lighting.

The tests were performed at the Ambient Intelligent Laboratory (AmILab), which had the area divided into four regions, with obstacles placed along the way, as shown in Figure 12. The audible alerts provided were recorded to assess the speed and accuracy of the presence and location of the obstacle. Users’ reactions were also recorded for each navigation (whether they obeyed or ignored the system alert).

The obstacles left in each region were as follows: region 1—wheelchair; region 2—brick with a height of 0.20 m; region 3—an air balloon hanging at 1.50 m in height; and region 4—a table with a height of 0.75 m. Table 6 shows the average error of users’ perceptions of obstacles in the horizontal and vertical planes.

**Figure 13:**
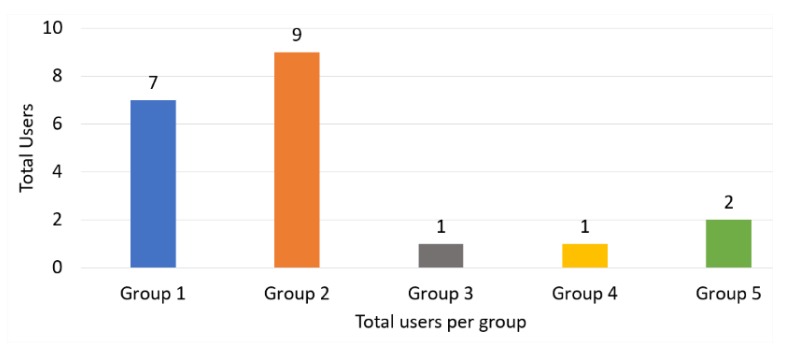
Cataloging collisions of reference group users.

A group of 20 visually impaired users (14 men and 6 women) participated in the tests to evaluate system behavior (algorithms and hardware). The age ranged from 26 years to 72 years. People had varying levels of vision, ranging from 10% of visual loss to totally blind. It was also identified that 16 people use a cane, while 4 do not use it to get around. These 20 users were initially identified based on their degree of visual impairment (Figure 13) and were divided into five groups: group 1—partial view, group 2—total loss of vision, group 3—total loss of vision associated with some cognitive/motor impairment, group 4—total vision loss associated with some physical disability, and group 5—others.

In order to make the tests homogeneous in the degree of difficulty, users were blindfolded to match those classified as total blind. Each user performed the test five times, totaling 100 tests. The procedures are as follows:

- Users were blindfolded to remove vision difference factor (partial blind and totally blind);
- To avoid information contamination, test users were interviewed and separated from the group.

**Figure 14:**
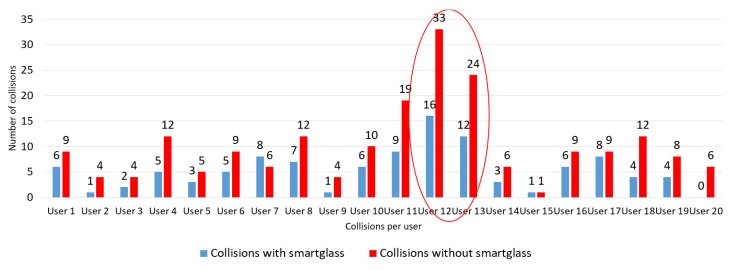
Cataloging of collisions by user.

All users were invited to travel the path plotted on the experimental route without the help of the electronic device the first time, as they defined it as most comfortable. Users then retraced the route using the device. The result of this test showed that the number of collisions with the obstacles is smaller when the smart glasses were used, as shown in Figure 14. Two users (user 12 and user 13) showed high difficulty performing the route without the device and with the device. Users described in their interviews that the loss of their visual abilities was accompanied by the loss of other senses, such as hearing (user 12) and some motor skills (user 13).

The navigation system test ends with the application of a questionnaire, where the user describes his feedback regarding the physical part (wearable device) and the logical part (the type of sound information, speed, hits, errors, and so on). A set of six questions were evaluated according to the Likert scale, following the relationship: 1—excellent, 2—very good, 3—good, 4—satisfactory, and 5—bad.

The interview questions were as follows: question 1—the quality of guidance; question 2—providing independence to users; question 3—identification of user position and obstacles; question 4—reliability generated by the use of the device; question 5—response time and question 6: system usability. Response percentages are cataloged in Table 7.

**Figure 15:**
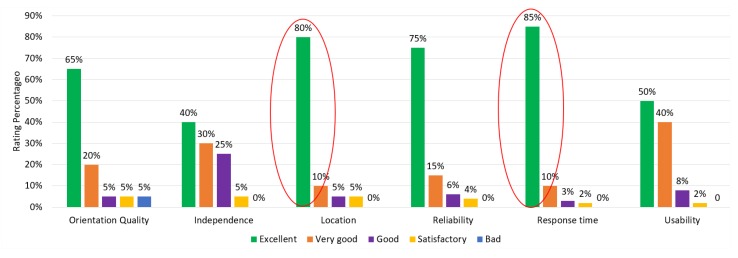
Detailed score of user-rated items.

Looking at the results of the assessment, the two factors with the best scores are response time with 85% and location with 80%. The rating indicated as excellent (green color) is highlighted in the graph in Figure 15.

**Figure 16:**
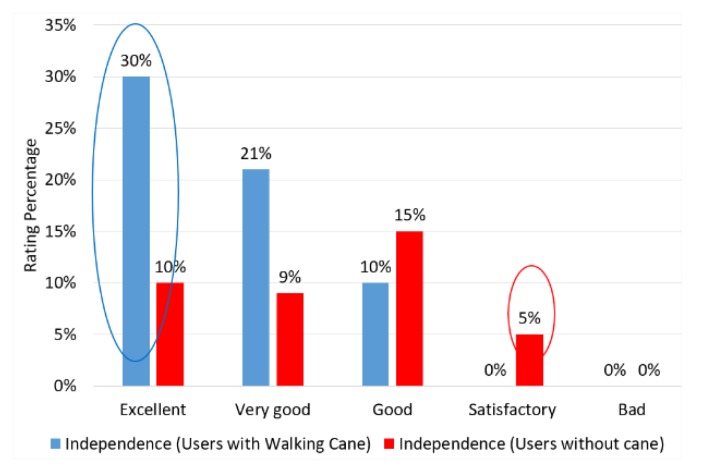
Detailed assessment of the two worst user-rated items.

The factor that got the worst score was independence, with only 40% of the excellent rating. This note represents the amount of user request for help to reposition in the scenario. The note for this item was opened so that a more detailed analysis could indicate its causes. Thus, users were organized into two groups: users with a cane, represented by the color blue, and users without a cane, represented by the color red. The results are depicted in the graph of Figure 16.

The results show that 30% of users who use a cane had less difficulty wearing smart glasses, rating the experience as excellent, while only 10% of users who do not use a cane can be rated excellent. A small group equivalent to 5% of the total users who do not use a cane showed a great difficulty in the experiment, evaluating the experience only as satisfactory.

**Figure 17:**
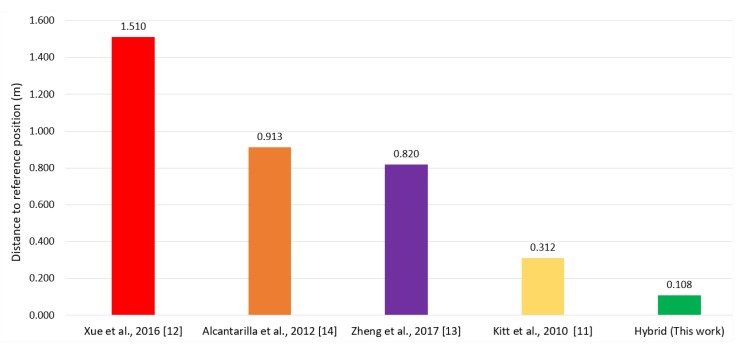
Comparison of the margin of error of the hybrid model and the related works.

The evaluative process is complex, and to be fair with the works used, it is crucial to use the same setup (equipment, software, and database). In spite of this, it was still possible to evaluate with a set of works that brought in their publications the information related to the algorithms, hardware, and databases, as well as the results obtained regarding the margins of error and the information delivery time. The system was confronted with a set of works to evaluate the impact of the choices used. Figure 17 graphically shows a comparative evaluation of these related works and this study in question.

Compared with Xue’s work, the proposed model showed more stable results. This stability is the direct result of applying a dynamic data reduction by the RANSAC algorithm, which determined a collection time, not a fixed amount of data volume [12]. The proposed model applied a distance calculation approach for obstacles by stereo vision, and unlike the model presented by Alcantarilla, there was a concern to indicate viable paths between the detected objects closest to the user [14]. The strategy adopted to identify the presence of obstacles was not concerned with describing what these obstacles were, as did Zheng, who developed visual markers and used a map to relate markers, increasing the complexity of his system [13]. The model built by Kitt also used visual markers; however, it had strong limitations of use in low light environments and with the different viewing angles of the floor marker [11]. The i-PDR algorithms and stereo vision obstacle detector stabilized location error margins and quickly provided accurate information on distances and obstacle positions and possible viable routes.

## 5. Conclusions and Future Work

This work proposed the construction of a hybrid indoor positioning system capable of being used in environments with distinct characteristics, such as open and unobstructed spaces and enclosed spaces with obstacles. The system combined two RGB sensors, data, and algorithms to increase accuracy without affecting the maximum delivery time.

In this model, two algorithms played an important role: the i-PDR and the 3D visual obstacle identifier. The i-PDR minimized the error margins of the PDR algorithm by using the Kalman filter and allowed safer and more reliable indoor navigation. The stereo vision obstacle identifier calculated the distance between the user and the obstacles in a pair of matrices representing the ground and the front of the user, indicating a viable path.

Another aspect considered important after performing this work is to know the physical elements used in the construction of the physical device. Both questions were perceived as dimensions and weight, as well as the perceptions and opinions of the test users. Although new mobile devices have shown higher processing power and memory, hybrid internal positioning algorithms still require resources, which may exceed the available physical limits. Indoor positioning systems should have architecture and robustness close to the already consolidated outdoor model, making it a useful resource for various audiences and problem classes [5].

This study has thrown a new look at the problem described in several localization and navigation projects in an indoor environment; that is, how to provide more accurate and reliable information to visually impaired users about the horizontal and vertical position of an obstacle in indoor navigation systems?

Briefly, the answers obtained were as follows:

(1) Accuracy is inversely proportional to time (velocity). That is, the more accurate the system, the more complex the algorithmic approach and the more time consumed, and vice versa. It is necessary to define the objective of the system (precision, speed), or to find a middle ground in the relations between the two factors.
(2) The data collection time is a crucial factor in the viability of indoor navigation. The time taken to collect and identify a candidate position should be as short as possible so that information is delivered within tolerable insurance limits to guide the user.
(3) The combination of musical beeps and spoken instructions enables the user to avoid obstacles more accurately and provide a more efficient guide for safer routes.

The use of stereo vision introduces to the study the first concern to keep the system stable in the relationship between accuracy and processing time. Thus, the strategy used was to submit the data to an intermediate solution in linearized algorithms.

Research on IPS has been driven by the pursuit of better levels of accuracy, creating new possibilities for user interaction with their indoor environments. Thus, this work also brings its contributions.

Among the contributions, a scheme of identification of regions in macro and microregions was presented, which reduces the understanding of the scenario by the obstacle identification algorithms and allows more verifiable navigation.

The combination of visual data in two vertical and horizontal matrices improved ground-level perception and facilitated the understanding of direction changes, indicating lines and curves to be followed by users.

The proposed hybrid model presented robust results when compared with mono visual location systems. The hybrid system also showed less position variation, providing a stable average accuracy, around 0.11 m.

This paper also introduced an obstacle detection approach by combining a stereo-visual measurement system and a sound scheme on the A + scale and C + scale. In this method, the stereo vision system extracts the protruding regions from the obstacles arranged in front of the user, triggering the sound corresponding to the suspect visual region. The tests performed using the stereo vision show that this method can detect obstacles effectively and correctly. However, by offering a new audio language, a training process is needed to reduce users’ learning time and make the proposed solution more user-friendly to the visually impaired.

## Figures and Tables

**Table 1:** Indication of the approaches presented. RANSAC, random sample consensus; KNN, K-nearest neighbors algorithm; PDR, pedestrian dead reckoning.

Author	Navigation Algorithms	Data Fusion	Alert Type
HEYA et al., 2018 [10]	SLAM	KNN	Sound
KITT et al., 2010 [11]	Proximity Method	Kalman filter	Visual
XUE et al., 2016 [12]	Proximity Method	RANSAC	Visual
PRESTI et al., 2019 [15]	Proximity Method	Weighted average	Polytonic
MASSICETI et al., 2018 [16]	Proximity Method	KNN	humming sound
BUJACZ et al., 2016 [17]	Proximity Method	Particle filter	humming sound
ALCANTARILLA et al., 2012 [14]	SLAM	Weighted average	Visual
CHEN et al., 2014 [6]	PDR	Kalman filter	Visual

**Table 2:** Set of instructions for indoor navigation.

Action	Answer of the Audio Guide
Drive forward	Go ahead
Turn right	Turn right on X meters
Turn left	Turn left on X meters
Turn right immediately	Turn right
Turn left immediately	Turn left
Alert: Close obstacle	Stop! Obstacle detected

**Table 3:** Mean of the margins of error presented by the visual and hybrid subsystems.

Location Strategy	Error Margin (m)
Visual Marker	0.454
Hybrid Marker	0.108

**Table 4:** Relation of the time factor and the use of subsystems.

IPS Type	Time (s)
Location for visual information	0.17
Hybrid location	0.07

**Table 5:** The relation between the number of frames processed and time.

Technique	Frames Per Second (FPS)
Image stereo	9
Image stereo with RANSAC	20
Image stereo, RANSAC, and particle filter	23

**Table 6:** Obstacle detection result.

Region	Height (m)	Distance (m)
Region 1	0.101	0.212
Region 2	0.205	0.647
Region 3	0.942	0.303
Region 4	0.942	0.129

**Table 7:** Results of questions after experiments.

Question	Performance Level				
	Excellent	Very Good	Good	Satisfactory	Bad
Orientation	65%	20%	5%	5%	5%
Independence	40%	30%	25%	5%	0%
Location	80%	10%	5%	5%	0%
Reliability	75%	15%	6%	4%	0%
Response time	85%	10%	3%	2%	0%
Usability	20%	65%	10%	5%	0%

## References

[1] Mainetti L., Patrono L., Ilaria S. A survey on indoor positioning systems. Proceedings of the 2014 22nd International Conference on Software, Telecommunications and Computer Networks (SoftCOM).

[2] Zengke L., Wang R., Gao J., Wang J. (2018). An Approach to Improve the Positioning Performance of GPS/INS/UWB Integrated System with Two-Step Filter. Remote Sens..

[3] Zhu Y., Mottaghi R., Kolve E., Lim J.J., Gupta A., Fei-Fei L., Farhadi A. Target-driven visual navigation in indoor scenes using deep reinforcement learning. Proceedings of the IEEE International Conference on Robotics and Automation (ICRA).

[4] Bayro Kaiser E., Lawo M. Wearable Navigation System for the Visually Impaired and Blind People. Proceedings of the IEEE/ACIS 11th International Conference on Computer and Information Science.

[5] Alatise M., Hancke G. (2017). Pose Estimation of a Mobile Robot Based on Fusion of IMU Data and Vision Data Using an Extended Kalman Filter. Sensors.

[6] Chen C., Chai W., Wang S., Roth H. (2015). A single frame depth visual gyroscope and its integration for robot navigation and mapping in structured indoor environments. J. Intell. Robot. Syst..

[7] Li X., Wang J., Liu C. (2016). Heading Estimation with Real-time Compensation Based on Kalman Filter Algorithm for an Indoor Positioning System. ISPRS Int. J. Geo-Inf..

[8] Yulong H., Yonggang Z., Ning L., Lin Z. (2015). Particle filter for nonlinear systems with multiple steps randomly delayed measurements. Electron. Lett..

[9] Chuang R., Jianping L., Yu W. Map navigation system based on optimal Dijkstra algorithm. Proceedings of the IEEE 3rd International Conference on Cloud Computing and Intelligence Systems.

[10] Heya T., Arefin S., Chakrabarty A., Alam M. Image Processing Based Indoor Localization System for Assisting Visually Impaired People. Proceedings of the Ubiquitous Positioning, Indoor Navigation and Location-Based Services (UPINLBS).

[11] Kitt B., Geiger A., Lategahn H. Visual odometry based on stereo image sequences with RANSAC-based outlier rejection scheme. Proceedings of the IEEE Intelligent Vehicles Symposium.

[12] Xue H., Ma L., Tan X. A fast visual map building method using video stream for visual-based indoor localization. Proceedings of the International Wireless Communications and Mobile Computing Conference (IWCMC).

[13] Zheng Y., Shen G., Li L., Zhao C., Li M., Zhao F. (2017). Travi-Navi: Self-Deployable Indoor Navigation System. IEEE/ACM Trans. Netw..

[14] Alcantarilla P., Yebes J., Almazán J., Bergasa L. On Combining Visual SLAM and Dense Scene Flow to Increase the Robustness of Localization and Mapping in Dynamic Environments. Proceedings of the IEEE International Conference on Robotics and Automation.

[15] Presti G., Ahmetovic D., Ducci M., Bernareggi C., Ludovico L., Baratè A., Avanzini F., Mascetti S. WatchOut: Obstacle Sonification for People with Visual Impairment or Blindness. Proceedings of the ASSETS ‘19 The 21st International ACM SIGACCESS Conference on Computers and Accessibility.

[16] Massiceti D., Hicks S., Rheede J.J. (2018). Stereosonic vision: Exploring visual-to-auditory sensory substitution mappings in an immersive virtual reality navigation paradigm. PLoS ONE.

[17] Bujakz M., Strumillo P. (2016). Sonification: Review of Auditory Display Solutions in Electronic Travel Aids for the Blind. Arch. Acoust..

[18] Skulimowski P., Owczarek M., Radecki A., Bujacz M., Rzeszotarski D., Strumillo P. (2018). Interactive sonification of U-depth images in a navigation aid for the visually impaired. J. Multimodal User Interfaces.

[19] Kumar S., Kumar P., Pandey S. Fast integral image computing scheme for vision-based applications. Proceedings of the 4th IEEE Uttar Pradesh Section International Conference on Electrical, Computer and Electronics (UPCON).

[20] Kalra A., Chhokar R.L. A Hybrid Approach Using Sobel and Canny Operator for Digital Image Edge Detection. Proceedings of the 2016 International Conference on Micro-Electronics and Telecommunication Engineering (ICMETE).

[21] Chanama L., Wongwitat O. A comparison of decision tree-based techniques for indoor positioning system. Proceedings of the IEEE International Conference on Information Networking (ICOIN).

[22] Liu T., Zhang X., Li Q., Fang Z. (2017). A Visual-Based Approach for Indoor Radio Map Construction Using Smartphones. Sensors.

[23] Zhou Y., Chen H., Huang Y., Luo Y., Zhang Y., Xie X. An Indoor Route Planning Method with Environment Awareness. Proceedings of the IGARSS 2018—2018 IEEE International Geoscience and Remote Sensing Symposium.

[24] Krause J., Perer A., Bertini E. (2014). INFUSE: Interactive feature selection for predictive modeling of high dimensional data. IEEE Trans. Vis. Comput. Graph..

[25] Chen C., Yang B., Song S., Tian M., Li J., Dai W., Fang L. (2018). Calibrate Multiple Consumer RGB-D Cameras for Low-Cost and Efficient 3D Indoor Mapping. Remote Sens..

[26] Song X., Wang M., Qiu H., Luo L. (2018). Indoor Pedestrian Self-Positioning Based on Image Acoustic Source Impulse Using a Sensor-Rich Smartphone. Sensors.

[27] Zhangaskanov D., Zhumatay N., Ali H. Audio-based Smart White Cane for Visually Impaired People. Proceedings of the 2019 5th International Conference on Control, Automation and Robotics (ICCAR).

[28] Spagnol S., Hoffmann R., Herrera Martínez M., Unnthorsson R. (2018). Blind wayfinding with physically-based liquid sounds. Int. J. Hum. Comput. Stud..

